# Central retinal artery occlusion without cherry-red spots

**DOI:** 10.1186/s12886-023-03176-w

**Published:** 2023-10-25

**Authors:** Wei Fan, Yanming Huang, Yuancheng Zhao, Rongdi Yuan

**Affiliations:** Department of Ophthalmology, The Second Affiliated Hospital of Army Medical University, 183th, Xinqiao street, Shapingba District, 400037 Chongqing, China

## Abstract

**Background:**

Cherry-red spots are a very important sign for the clinical diagnosis of central retinal artery occlusion (CRAO). We retrospectively summarized the clinical manifestations of CRAO and analysed the causes and characteristics of CRAO without cherry-red spots. In this study, we explored a diagnostic method for CRAO without cherry red spots.

**Methods:**

Seventy patients (70 eyes) with CRAO were examined retrospectively. Corrected distance visual acuity, fundus photos, FA and OCT images were collected at the first outpatient visit. The causes of CRAO without cherry-red spots were analysed through fundus photos. The incidence of increased hyperreflectivity of the inner retina, central macular thickness (CMT) and arteriovenous transit time in patients with and without cherry-red spots were compared.

**Results:**

Fundus examination showed posterior retinal whitening in 57 cases (81.43%) and cherry-red spots in 39 cases (55.71%). Thirty-one patients presented at the first outpatient visit without cherry-red spots. The reasons for the absence of cherry-red spots included leopard fundus (32.26%), retinal vein occlusion (25.81%), no obvious inner retinal coagulative necrosis (19.35%), ciliary retinal artery sparing (12.90%), high macular oedema (9.68%) and cherry-red spot enlargement (3.23%). OCT revealed increased hyperreflectivity of the inner retina in 67 CRAO patients (95.71%). All 3 patients without increased hyperreflectivity of the inner retina did not present with cherry-red spots at the first visit. The median CMT in patients without cherry-red spots was 166.00 μm, while the median MCT in patients with cherry-red spots was 180.00 μm; there was no significant difference between these two groups (P = 0.467). FA showed delayed arteriovenous transit time > 23 s in 20 patients (28.57%), > 15 s in 43 patients (61.43%) and no delay in 27 patients (30.77%). The median arteriovenous transit time in patients without cherry-red spots was 19.00 s, while it was 18.00 s in patients with cherry-red spots; there was no significant difference between these two groups (P = 0.727).

**Conclusions:**

There are multiple factors that could cause the absence of cherry-red spots in CRAO. The use of OCT to observe increased hyperreflectivity of the inner retina is the most effective imaging method for the early diagnosis of CRAO without cherry-red spots.

## Background

Central retinal artery occlusion (CRAO) is an ophthalmic emergency that results in a poor visual outcome [1]. A literature review showed that the window of treatment for total CRAO is 90–240 min [2]. Therefore, accurate diagnosis and timely management of CRAO are very important for improving patient prognosis.

Currently, fluorescein angiography (FA) remains the gold standard for diagnosing CRAO. However, FA is an invasive and time-consuming examination that takes at least 20 min. Twenty minutes might be just a moment for normal human beings, but it is a long time for someone who is experiencing retinal infarction. In addition, patients with a history of severe allergies, asthma and renal dysfunction cannot undergo FA [3]. A recent study of CRAO by Abdellah MM showed a delayed arteriovenous transit time > 23 s in 53.33% of cases and normal FA results in 26.67% of cases [4], thus providing a challenge to its role in diagnosing CRAO.

The clinical diagnosis of CRAO depends on history and funduscopic examination. However, CRAO associated with normal-appearing retinal vessels and observable emboli is not rare. Among the funduscopic findings of CRAO [5], cherry-red spots are the most recognizable and typical sign. In CRAO, the reddish color of the vascular choroid and pigment epithelium is still seen through the foveola, which is surrounded by an area of the white/opacified retina; this gives rise to the typical cherry-red spot. Cherry-red spots usually become evident after several hours of ischaemia and usually persist for several days after CRAO. In most cases, the diagnosis of CRAO can be made by assessing patient history and obtaining fundoscopic findings of cherry red spots. The critical time from onset of artery occlusion to inner retinal infarction represents a window for emergency treatment to prevent catastrophic visual loss [2]. Rapid clinical diagnosis of CRAO helps to quickly implement therapeutic interventions within the window and maximize the recovery of patients’ visual function. The absence of cherry-red spots is a challenge for the diagnosis of CRAO at the first visit and might delay various treatments.

However, cherry-red spots are not unique to CRAO. Diseases that lead to retinal ischaemia and infarction and as well as diseases that lead to the accumulation of different substances in retinal ganglion cells might also present with cherry-red spots [6–8]. In addition, cherry-red spots do not occur in all CRAO patients, especially in the chronic stage and during reperfusion of the retinal vessels, when the fundus colour returned to normal. The incidence of cherry-red spots in CRAO is inconsistent. A recent study reported that cherry-red spots were present in 66.7% of CRAO patients [4]. In the present study, we retrospectively collected the clinical manifestations of 70 cases of CRAO and analysed the causes and imaging features of CRAO without cherry-red spots.

## Methods

Seventy eyes (seventy patients) diagnosed with CRAO in the Second Affiliated Hospital of the Army Medical University from November 2015 to July 2022 were retrospectively studied. The ethics committee of the Second Affiliated Hospital of the Army Medical University approved this study. The study registration number in the China Clinical Trial Center is ChiCTR2200061913.

All methods were performed in accordance with the Declaration of Helsinki and relevant clinical trial management regulations of China. The diagnosis of CRAO was made based on the history of sudden monocular vision loss, posterior retina whitening and cherry-red spots in fundus examination, delayed arterial filling on FA and hyperreflectivity of the inner retina on optical coherence tomography (OCT) [9]. All patients with inadequate information on history and fundus findings or ambiguous diagnosis were excluded. In addition, CRAO patients with obvious evidence of giant cell arteritis were also excluded. The following data were collected at the first outpatient visit: best corrected visual acuity (BCVA), fundus photo, OCT images (Zeiss cirrus HD-OCT, Germany and TOPCON Triton SS-OCT, Japan) and FA images (Heidelberg spectroscopy HRA, Germany). The incidence of increased hyperreflectivity of the inner retina was separately judged by two experienced ophthalmologists, and if the results were consistent, they were included in the statistical analysis. Any inconsistencies were resolved by consulting a third senior physician. Central macular thickness was manually measured using a built-in measurement system, with the measurement standard being the distance from the inner limiting membrane of the macular centre to the pigment epithelial layer. Two physicians measured it separately, and the measurement results were consistent through intragroup correlation coefficient analysis (ICC = 0.998, P < 0.01). Finally, one of the measurement results was used. The arteriovenous transit time was directly obtained through FA.

Fundus manifestations were statistically analysed using SPSS 22.0 (SPSS Inc., Chicago, Illinois, USA).Quantitative data are expressed as the means ± standard deviations (SD). Qualitative data are expressed as numbers and percentages. The data were tested for normality using the Shapiro‒Wilk test. Independent samples t tests were used for normally distributed data. A P value below 0.05 was considered statistically significant.

## Results

### Demographics

Seventy patients (seventy eyes) were included in the present study. The median age was 61 years old, ranging from 19 to 83. The study included 48 males (68.6%) and 22 females (31.4%). All CRAO patients had visual acuity lower than 20/200, 11 patients (15.71%) had visual acuity between 20/2000 and 20/200, 59 patients (84.29%) had visual acuity lower than 20/2000, and 5 patients (7.14%) had no light perception. The time from initiation of symptoms to the first outpatient visit ranged from 1 h to 7 days with a medium time of 3 days, of which 6 patients (8.57%) had outpatient visits that were shorter than 4 h. Most CRAO patients had systemic diseases, with 41 cases (58.57%) of hypertension, 11 cases (15.71%) of diabetes, 4 cases (5.71%) of cerebral infarction, and 3 cases (4.29%) of coronary heart disease.

### Fundus characteristics of CRAO

In the present study, fundus changes in CRAO were located in the posterior retina, with a comparatively normal peripheral retina. Fundus characteristics, including posterior retinal whitening, cherry-red spots, optic disc oedema, haemorrhage, leopard-shaped fundus, cotton-wool spots and ciliary retinal artery perfusion, were recorded and analysed. The fundus characteristics of patients with CRAO are shown in Table 1. The three most common fundus characteristics were posterior retinal whitening (81.43%), cherry-red spots (55.71%), and optic disc oedema (37.14%). Seven patients (10%) had ciliary retinal artery perfusion.

**Table 1:** Fundus characteristics of CRAO

Fundus characteristics	Cases	Percentage
Posterior retinal whitening	57	81.43%
Cherry-red spot	39	55.71%
Optic disc oedema	26	37.14%
Haemorrhage	14	20%
Leopard print fundus	12	17.14%
Cotton wool spot	7	10%
Ciliary retinal artery perfusion	7	10%

### Reasons for CRAO without cherry-red spots

**Fig. 1:**
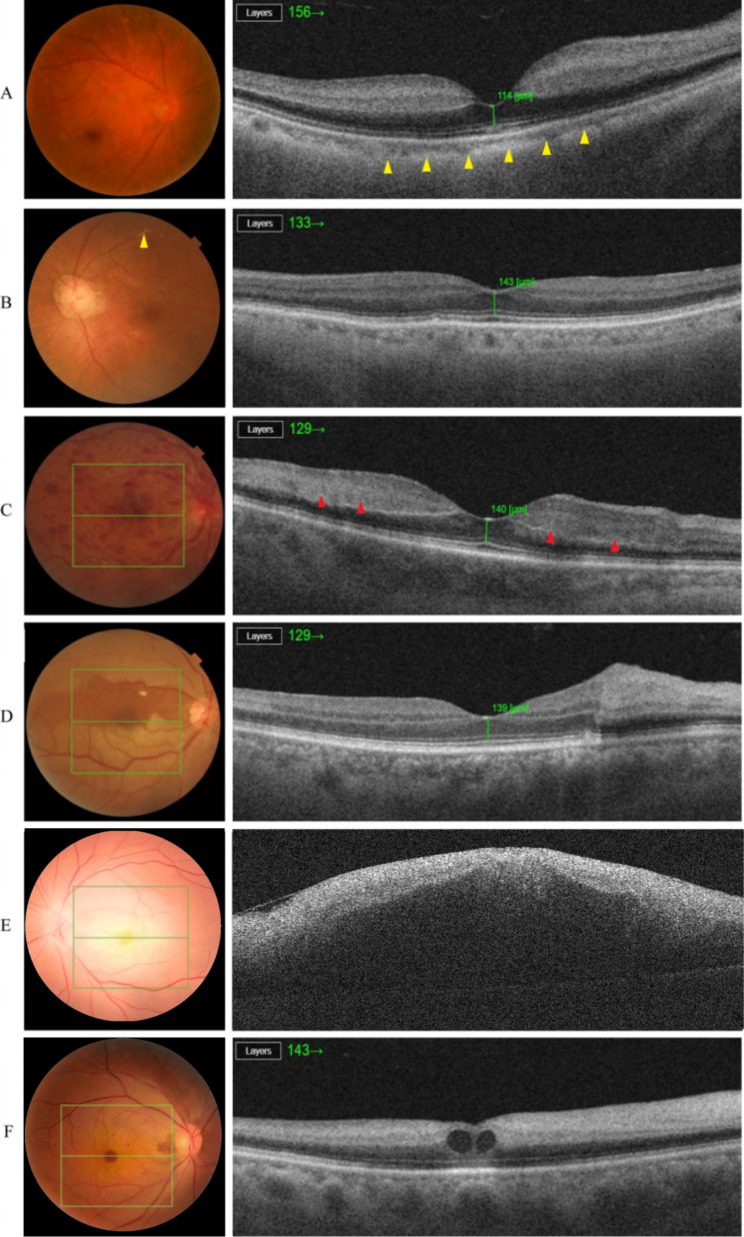
** A**. One CRAO patient with leopard print fundus showed no typical cherry-red spots. OCT indicated that the choroid was significantly thinner (yellow arrowheads). **B**. One patient presented 1 h after the onset of CRAO without cherry-red spots. Yellow‒white arterial emboli (the yellow arrowhead) could be seen in the superior temporal branch artery. OCT indicated that there was no obvious inner hyperreflexia. **C**. One CRAO patient combined with RVO showed no cherry red spots. Massive retinal haemorrhage could be seen in the fundus. OCT showed obvious inner retinal thickening and segmental increased reflection in the inner and outer reticular layers of the macular area (the red arrowheads) that manifested as acute para central middle maculopathy lesions. **D**. One CRAO patient with ciliary retinal artery perfusion showed no cherry red spots. OCT indicated a dividing line between the ciliary retinal artery perfusion area and infraction area. E. One CRAO patient with unknown macular oedema showed no cherry red spots. F. One CRAO patient with diabetes retinopathy showed no cherry red spots (the OCT scanning area was the centre of the macula). It was mistaken for macular haemorrhage. OCT showed cystoid macular oedema and obvious inner retinal thickening and high reflection

Cherry-red spots were present in 39 eyes (55.71%). The reasons for CRAO without typical cherry-red spots are shown in Table 2. Various diseases that have leopard print fundus are the primary reason for CRAO without cherry red spots, which accounts for 32.26% of the reasons. In Fig. 1A, one CRAO patient with leopard print fundus due to high myopia did not show typical cherry red spots. In this case, the choroid was significantly thinner than normal (indicated by the yellow arrow) due to atrophy of the small vessel layer. The inner retina showed high reflection and obvious oedema. Eight cases (25.81%) of CRAO combined with retinal vein occlusion (RVO) did not show typical cherry-red spots. In Fig. 1C, although the inner retinal thickening and high reflection are obvious, the CRAO patient did not show cherry-red spots in the fundus. Extensive flaming retinal haemorrhage due to RVO covered the fovea. In addition, various reasons that lessen the chance of manifesting inner retinal coagulative necrosis and high reflection (6 cases, 19.35%) could cause the absence of cherry red spots in CRAO. In Fig. 1B, one patient presented 1 h after the onset of CRAO with yellow and white arterial emboli (indicated by yellow arrows) at the posterior retina; the patient did not show typical signs of cherry-red spots. The reason is that the inner retinal coagulative necrosis and increased hyperreflective is not obvious, which could be confirmed by OCT, because the infarction time is too short. In the present study, 4 cases (12.90%) of CRAO with ciliary retinal artery sparing also led to the absence of cherry-red spots. In Fig. 1D, one CRAO patient with ciliary retinal artery sparing showed a clear dividing line between the ciliary retinal artery perfusion area and infraction area. OCT showed obvious inner retinal thickening and increased hyperreflective in the infarction area. In addition, 3 cases (9.68%) of CRAO with macular oedema and 1 case (3.23%) of CRAO with cherry-red spot enlargement did not show typical cherry-red spots. In Fig. 1D, one patient with CRAO did not show cherry-red spots due to high macular oedema, as shown by OCT. In Fig. 1E, a CRAO patient combined with unknown severe macular oedema showed no obvious cherry-red spots. In Fig. 1F, a CRAO patient combined with diabetes retinopathy, the cherry-red spot was actually enlarged after macular cystoid oedema due to diabetes retinopathy. It was mistaken for macular haemorrhage.

**Table 2:** Reasons for CRAO without cherry-red spots

Reasons	Cases	Percentage
Leopard print fundus	10	32.26%
Coexistence of RVO	8	25.81%
Inner retinal coagulative necrosis not obvious	6	19.35%
Ciliary retinal artery sparing	4	12.90%
Macular oedema	3	9.68%
Cherry red spot enlargement	1	3.23%

### Comparison of CRAO with or without cherry red spots

In patients with CRAO, intracellular oedema occurs in inner retinal cells due to ischaemia and hypoxia, which manifests as increased hyperreflectivity of the inner retina on OCT. Retinal cells are nerve cells that have high metabolism and high oxygen consumption. In the present study, 90.33% of CRAO patients without cherry red spots had high reflection of the inner retina, while all patients with cherry red spots had high inner retina reflection. The difference between these two groups was significant (Table 3, p = 0.047). With the progression of ischaemia, extracellular oedema develops in the posterior retina, including the macula, manifesting as typical posterior retinal opacity. The median central macular thickness in CRAO patients with and without cherry red spots was 180 μm and 166 μm, respectively. The difference between these two groups was not significant (Table 3, p = 0.467).

**Table 3:** Comparison of OCT characteristics between CRAO without cherry red spots and CRAO with cherry red spots

OCT characteristic	CRAO without cherry red spots		CRAO with cherry red spots		P value
	Cases/total cases	Percentage	Cases/total cases	Percentage	
Increased hyperreflectivity of the inner retina	28/31	90.33%	39/39	100.00%	*P* = 0.047
Central macula thickness (µm)	166.00(140.00,263.00)		180.00(156.00,237.00)		*P* = 0.467

Delayed arteriovenous transit time could be seen in most CRAO patients. The CRAO without cherry-red spots group had a higher proportion of patients with a normal arteriovenous transit time of ≤ 15 s (48.39% vs. 30.77%); however, due to the limited number of cases, this difference was not significant (p = 0.133). The proportion of patients with a prolonged arteriovenous transit time (i.e., > 23 s) was lower in the CRAO without cherry-red spots group (25.81% vs. 30.77%), but the difference was not statistically significant (Table 4, p = 0.648). The median arteriovenous transit times in CRAO patients with and without cherry red spots were 18 and 19 s, respectively; this difference was not significant (Table 4, p = 0.727).

**Table 4:** FA characteristic comparison of CRAO with or without typical cherry-red spots

FA characteristic	CRAO without cherry red spots		CRAO with cherry red spots		P value
	Cases/total cases	Percentage	Cases/total cases	Percentage	
Arteriovenous transit time <23s	8/31	25.81%	12/39	30.77%	*P* = 0.648
Arteriovenous transit time <15 s	16/31	51.61%	27/39	69.23%	*P* = 0.133
Arteriovenous transit time ≤ 15 s	15/31	48.39%	12/39	30.77%	*P* = 0.133
Arteriovenous transit time (s)	19.00 (13.00,23.00)		18.00 (14.00,23.00)		*P* = 0.727

## Discussion

CRAO is usually diagnosed based on the patient’s history and based on clinical findings of typical cherry-red spots. However, cherry-red spots are not a unique manifestation of CRAO. Various diseases that cause retinal opacity could also have cherry-red spots. In addition, some cases of CRAO may present without cherry-red spots, which makes the diagnosis challenging. The critical time from onset of artery occlusion to functionally significant inner retinal infarction represents a window for treatment and therapeutic interventions. The retina of monkeys can tolerate ischaemia for approximately 100 min without significant histological changes. Irreversible retinal damage occurs 240 min after infarction [10]. Currently, the optimal treatment time window for CRAO is widely believed to be 240 min. This study aimed to improve the diagnostic rate of CRAO at the first visit and gain valuable time for subsequent intervention.

A previous study by Hayreh et al. revealed that cherry-red spots were present in 90% of patients with permanent CRAO, while posterior retina opacity accounted for 58%, and optic disc oedema accounted for 22% [5]. Marwa et al. reported that cherry-red spots were present in 66.7% of CRAO patients [4]. The present study showed that posterior retina opacity was the most common ocular finding (81.43%), while cherry-red spots were present in 55.71% of CRAO patients. The reasons for the inconsistent incidence of cherry-red spots in CRAO patients were as follows: (1) The present study included CRAO with cilioretinal artery sparing. This subtype of CRAO was associated with a lower incidence of macular abnormalities. (2) In this study, the incidence of patients who presented with cherry-red spots at the first outpatient visit was calculated, while the incidence of cherry-red spots was obtained in the first week of presentation in Hayreh’s study.

The present study showed that the most common cause of CRAO without cherry-red spots is a leopard-shaped fundus (32.26%). In addition, venous obstruction, the lack of obvious inner retinal coagulative necrosis, ciliary retinal artery sparing, high macular oedema, and cherry-red spot enlargement could also lead to the absence of cherry-red spots. Due to the limited number of CRAO patients, it was not possible to identify all the potential reasons for the absence of cherry-red spots. Based on the mechanism of cherry-red spots, we inferred that the following circumstances that affect the contrast between the macula and posterior retina could cause the absence of cherry-red spots in CRAO patients: ① abnormalities in choroidal blood vessels include leopard-like fundus due to choroidal atrophy in high myopia patients (Fig. 1A) and elderly patients and choroidal ischaemia caused by occlusion of the ophthalmic artery or ciliary retinal artery; ② abnormalities in the pigment epithelium of the macula, such as pathological myopia, age-related macular degeneration, and Stargardt’s disease; ③ the thickness of the retina becomes thinner, which can be seen in patients with retinitis pigmentosa, advanced age, and late-stage glaucoma; ④ insufficient oedema or opacity of the inner retina (when patients came to the clinic shortly after retinal infarction or the central retinal artery was partially blocked, the inner retina had less obvious oedema, as seen in Fig. 1B); ⑤ various macular changes, such as macular haemorrhage, exudation, and oedema, can obscure the colour of the choroid (Fig. 1C, E, F); and ⑥ the macula is supplied by the ciliary retinal artery when the central retina is infarcted (Fig. 1D).

How to quickly and accurately diagnose patients with sudden vision loss who present without cherry-red spots at the first visit has become an urgent problem. Although FA is currently still the gold standard for diagnosing CRAO, the invasive and time-consuming nature of FA often delays the treatment of CRAO. In this study, we found that patients without cherry-red spots had a higher number of cases with normal arteriovenous transit time (< 15 s) and a lower number of cases with delayed arteriovenous transit time (> 23 s). However, due to the limited number of cases, there was no statistically significant difference compared to the patients with cherry-red spots. The proportion of delayed arteriovenous transit time in patients without cherry-red spots was 25.81%. Therefore, FA cannot be the preferred examination for diagnosing CRAO in patients without cherry-red spots.

In the present study, only 3 of the CRAO patients did not show increased hyperreflectivity of the inner retina on OCT, and they presented without cherry-red spots at the first visit. The incidence of increased hyperreflectivity of the inner retina in patients without cherry-red spots was 90.33%. Therefore, OCT is recommended as the preferred examination for diagnosing CRAO in patients without cherry-red spots. The reasons why these CRAO patients presented without increased hyperreflectivity of the inner retina and cherry-red spot might be obvious in the very early stage after retinal infarction, incomplete occlusion, presence of ciliary retinal blood supply, or recanalization after occlusion. We should be particularly cautious about these patients, and ocular coherence tomography angiography (OCTA), FA, or electrophysiological examinations should be performed to comprehensively evaluate the condition. OCTA is a noninvasive examination that can display the blood flow of the retina. Clear OCTA images were obtained in a few CRAO patients due to difficult fixation and long acquisition time. It was found that OCTA displayed disruption of the superficial and deep retinal capillary plexus in the early stage of CRAO. Retinal capillary plexus changes on OCTA appear earlier than increased hyperreflectivity of the inner retina on OCT [11]. However, most patients with CRAO have poor vision and have difficulty in fixation, which can affect the image quality of OCTA.

This study analysed possible reasons for the absence of cherry-red spots in CRAO and explored recommended examinations when CRAO is suspected without cherry-red spots. The limitation of this study is that it includes all types of CRAO, and different subtypes of CRAO might have different manifestations. Second, this study only obtained clear OCTA images in a minority of CRAO patients. OCTA can be used to detect early signs of retinal ischaemia, and the combination of OCT and OCTA is worth further research in the early diagnosis of CRAO without cherry red dots.

## Conclusions

CRAO without cherry-red spots is not rare. Patients with sudden vision loss but without cherry-red spots should be taken seriously at the first outpatient visit. Increased hyperreflectivity of the inner retina by OCT is a fast and noninvasive diagnostic examination with high specificity for CRAO. The increasing application of OCTA might be useful for the early diagnosis of CRAO without cherry-red spots.

## Data Availability

The datasets used and/or analysed during the current study are available from the corresponding author on reasonable request.

## Abbreviations

CRAO: central retinal artery occlusion
CMT: central macula thickness
FA: Fluorescein angiography
OCT: Optical coherence tomography
RVO: retinal vein occlusion
OCTA: ocular coherence tomography angiography
